# Association between prenatal exposure to ambient ozone, birth weight, and macrosomia in healthy women

**DOI:** 10.3389/fpubh.2022.1000269

**Published:** 2022-11-07

**Authors:** Chengyi Zheng, Jiaqi Tian, Lan Ma, Chunjie Ding, Lin Zhang

**Affiliations:** ^1^Qihe Maternal and Child Health Care Hospital of Shandong Province, Dezhou, China; ^2^Clinical Medical Research Center for Women and Children Diseases, Maternal and Child Health Care Hospital of Shandong Province Affiliated to Qingdao University, Jinan, China

**Keywords:** birth weight, macrosomia, ozone, generalized addictive model, birth outcome

## Abstract

Studies have shown that prenatal ozone exposure is associated with an increased risk of adverse pregnancy outcomes, among which abnormal birth weight is a detrimental factor for diseases in adulthood, but the association between birth weight and ozone is inconclusive. Herein, we conducted this study by enrolling 407 couples of pregnant women and collected their demographical materials, their exposure to ambient ozone was assessed according to the place of their residence. The hourly monitored ozone was first averaged to the daily level, then monthly and whole-gestationally levels. After adjusting confounders, we processed a multivariate generalized addictive analysis to predict the association between prenatal ozone exposure and birth weight. We also divided the cohort into two categories according to whether the infant met the standard of macrosomia, and the occurrence of macrosomia was studied via univariate and multivariate logistic regression analyses as extreme conditions of the effects of ozone exposure on birth weight. We found that the ground-level ozone in Jinan changed with temperature periodically, higher in summer and lower in winter. Over the past 8 years from 2014, the ambient ozone increased by 1.74 μg/m^3^ per year. Of the 407 singleton-pregnant women, 21 infants were diagnosed with macrosomia. After adjusting confounders, we found that each unit increase in prenatal ozone exposure caused 8.80% [OR_ozone_90%CI: 0.912 (0.850, 0.978)] decreased risk of macrosomia, but the splined ambient ozone exposure data was not statistically associated with birth weight, which is probably due to the limited sample size. In conclusion, prenatal ozone exposure is associated with decreased risk of macrosomia but is weakly linked to birth weight.

## Introduction

Birth weight has long been studied as a crucial indicator reflecting the health status of the newborn, and it is also recognized as an indicator potentially associated with many diseases in adulthood. Low birth weight (LBW), for example, is defined as a newborn weighing <2,500 g, for whom there are increased risks of perinatal morbidity and cardiovascular and metabolic diseases in adulthood (1, 2). In contrast, macrosomia refers to a newborn with bodyweight >4,000 g regardless of gestational age, and it is commonly associated with fetal asphyxia, shoulder dystocia, birth trauma, and neonatal hypoglycemia (3–5), but also adverse sequelae such as diabetes, high blood pressure, and obesity in childhood and adulthood (6, 7). Over the past decades, researchers have revealed dozens of risk factors leading to lower or higher birth weight, but the etiology of birth weight dysregulation is still not all known.

Over recent years, ground-level ozone (O_3_) has intrinsically increased with climate change, and ozone pollution is gradually becoming an essential constituent of air pollution which challenges the physical inner homeostasis of human beings. Generally, ozone in the air is not directly emitted but is generated by photochemical reactions in the troposphere, in which sunlight and ozone-precursors such as nitrogen oxides (NO_x_), volatile organic compounds (VOCs), and carbon monoxide (CO) are involved. Driven by climate change, the rate of temperature change often accelerates along with altitude rises, and temperatures above 4,000 meters have warmed 75 percent faster than at altitudes below 2,000 meters over the past 20 years (8). However, it has been demonstrated that temperature can directly influence ozone production by accelerating the rate of chemical reactions and increasing the emissions of VOCs from vegetation (9).

Ozone in the lower troposphere is a strong oxidant, and elevated ozone at this layer is harmful to materials and nearly all organisms on earth. By definition, ground-level ozone is also a reactive oxidant gas that primarily constitutes atmospheric smog. To date, the main known health concern of ground-level ozone exposure is respiratory disorders, which is supported by a large number of studies (10). As far back as 1989, WHO reports indicate that short-term exposure to ground-level ozone between 200 and 500 μg/m^3^ induces temporary eye and respiratory irritation shown as cough, chest discomfort, and headache (11). Recently, acute ozone exposure is further demonstrated to cause inflammation in the neutrophilic airway and transient reduction in lung function (12). In China, there are 318 cities with ozone exceeding the WHO standard, and 74, 233 of total premature mortality with 7.6 billion $ lost/year should be attributed to ozone exposure (13). Based on a randomized clinical trial, Michelle L qualified the adverse effect of ozone exposure on lung functions, they reported that exposure to 70 ppb O_3_ for 6.6 h could result in an absolute decrease of 1.8 ± 0.5% in % predicted forced vital capacity (14). Moreover, results from two German cohort studies suggest that short-term exposure to ozone causes an oxidative stress response in the skin, and long-term ozone exposure is associated with matrix metalloproteinase upregulation, resulting in premature human skin aging (15, 16).

The ground-level ozone is formed naturally by chemical reactions of NOx gases with volatile organic compounds (VOCs) under sunlight, and ozone in the troposphere occupies 10% of the total number of ambient ozone (17). Commonly, tropospheric ozone is considered a greenhouse gas threatening human health, and contributes to global warming. Ambient ozone can be measured through remote sensing technology and *in-situ* monitoring technology (18), for data we utilize in this study, it is acquired from the air quality environmental monitoring networks, where, based on ozone's UV-absorption properties, the *in-situ* ozone monitors are applied to measure ppb-levels of ambient ozone. Over the past years, the variation of ambient ozone and its adverse health effects have been drawing considerable attentions. For example, due to the increase in anthropogenic emissions, researchers have found a particularly increasing trend of tropospheric ozone in the northern hemisphere. In contrast, increases of ozone concentration over Europe and North America appear to be slight (19). In Antarctica, based on >25 years of surface ozone and ozonesonde measurement data, Pankaj and colleagues report that a positive trend in ozone exists at the surface and lower and mid-troposphere, and the authors alert the increase of Antarctic ozone and its warming feedback to the Earth's climate (20).

For human beings, ozone is a well-defined oxidizing agent, and breathing ground-level ozone can result in a series of adverse health effects, including induction of respiratory symptoms, decrements in lung function, and inflammation of airways (21, 22). Notably, respiratory symptoms are widely studied, commonly shown as coughing, throat irritation, and pain, burning, or discomfort in the chest when taking a deep breath (23). Prenatal exposure to ozone is linked to an increased risk of childhood wheezing, the second trimester is supposed to be the susceptible exposure window. Moreover, third-trimester ozone exposure is indicated to be associated with a 20% increased risk of intrauterine growth retardation (95%CI = 1.0–1.4) (24). Nonetheless, the association between prenatal ozone exposure and birth weight is still inconclusive. Most recently, maternal ozone exposure is reported to be associated with an increased risk of term low birth weight (25), but results concerning the qualification of ozone-induced birth weight reduction are absent.

To investigate the impacts of prenatal ambient ozone exposure on birth weight, we analyzed the variation of atmospheric ozone levels over the last 8 years, based on which we established statistical models toward the occurrence of macrosomia as extreme conditions. These results would facilitate the risk qualification of prenatal ozone exposure on birth weight and contribute to its intervention.

## Methods

### Population

The study protocol was reviewed and approved by the Ethics Committee of Maternal and Child Health Care Hospital of Shandong Province, Jinan, China. The information used in this study was anonymous, and no individually identifiable records exist.

This study was adopted in the Maternal and Child Health Care Hospital of Shandong Province, where 407 couples with singleton birth pregnant women from 2018 to 2019 were enrolled. The details of the subjects were collected from the electronic medical record system and questionnaires, including maternal age, pre-pregnancy body mass index (BMI), height, weight, age at menarche, menstrual status, education background, history of gestation, parity, abortion, and live birth, paternal smoking and drinking status, gestational age, sex of the newborn, and fetal weight. The criteria for excluding samples were traveling out over 30 days during pregnancy, being conceived by human-assisted reproduction techniques, being diagnosed with a congenital disability at the neonatal screening stage, and conceived at <18 or >45 years old. The missing values of the final dataset were randomly imputed by the *MICE* package in R software.

### Outcome definition

According to birth weight, the infants are divided into three categories: the normal birth weight group includes babies born weighing between 2,500 and 4,000 g, low birth weight is defined as a baby born weighing <2,500 g, and macrosomia refers to birth weight beyond 4,000 g regardless of gestational age.

### Exposure assessment

In line with our previous study (26), the hourly monitored atmospheric ozone in Jinan is acquired from the Air Quality and Pollution Measurement platform, publicly available at https://aqicn.org/. The data were collected from 2014 to 2021, during which the prenatal ozone exposure level is calculated based on the daily averaged ozone from the first day of pregnancy confirmation to the day of delivery. In parallel, the meteorological parameters of local temperature and wind direction and speed were acquired from the NOAA Integrated Surface Database (ISD), available through *worldmet* package in R software. The long-term trend of atmospheric ozone level was analyzed through Theil–Sen method.

### Covariates

According to previous studies (27), we collected data on birth weight-related demographical variables, and their potential effects on birth weight were compared and adjusted through multivariate analyses. These confounders include maternal age, pre-pregnancy body mass index (BMI), age at menarche, menstrual status, educational background, history of gestation and parity, paternal smoking and drinking status, gestational age, and sex of the newborn. Among which maternal age, BMI, age at menarche, and gestational age were involved as continuous variables, menstrual status, history of gestation and parity, paternal smoking and drinking status, and sex of the newborn were binary variables. Maternal education background was divided into three categories of primary, middle, and senior levels. The hourly monitored atmospheric ozone was collected then transformed to daily levels and finally averaged to pregnancy levels.

### Statistics

All demographical variables of the study subjects and their corresponding atmospheric ozone exposure levels were integrated into one dataset. The continuous variables were represented as mean and standard deviation, the categorized variables were shown as frequency with correlated percentage. According to birth weight, we divided the cohort into two groups, where the macrosomia group were infants weighing ≥4,000 g, and the non-macrosomia group were babies weighing <4,000 g. The differences between these two groups were compared through a *t*-test for continuous variables and a chi-square test or Fisher exact probability test for categorized variables.

Three statistical models were built to evaluate the effect of ozone exposure on birth weight, where macrosomia was set as a dependent variable in logistic regression analyses, and birth weight was employed as a continuous variable in the generalized additive model. In detail, the univariate logistic analysis was conducted based on the prenatal ozone exposure level and confounders of birth weight, each time one factor was independently involved in the statistical model. By simultaneously enrolling the prenatal ozone exposure data and all confounders of birth weight in one model, the multivariate analysis was built to adjust the bias of demographical variables, and only items with *p*-value <0.05 were reserved. Also, the area under the receiver operator characteristic curve (AUC) was generated to test the robustness of the results of multivariate logistic regression analysis. Finally, the generalized additive model analysis was conducted to explore the linearity of ozone exposure and birth weight, where all demographical variables and ozone exposure data were involved, of which BMI and ozone were shown as splined items.

The visualization, description, comparison, and modeling of data were processed using R software version 4.1.0 for Windows. The differences between groups of *p*-value <0.10 were considered statistically significant unless otherwise indicated.

## Results

### Characterization of atmospheric ozone in Jinan

**Figure 1:**
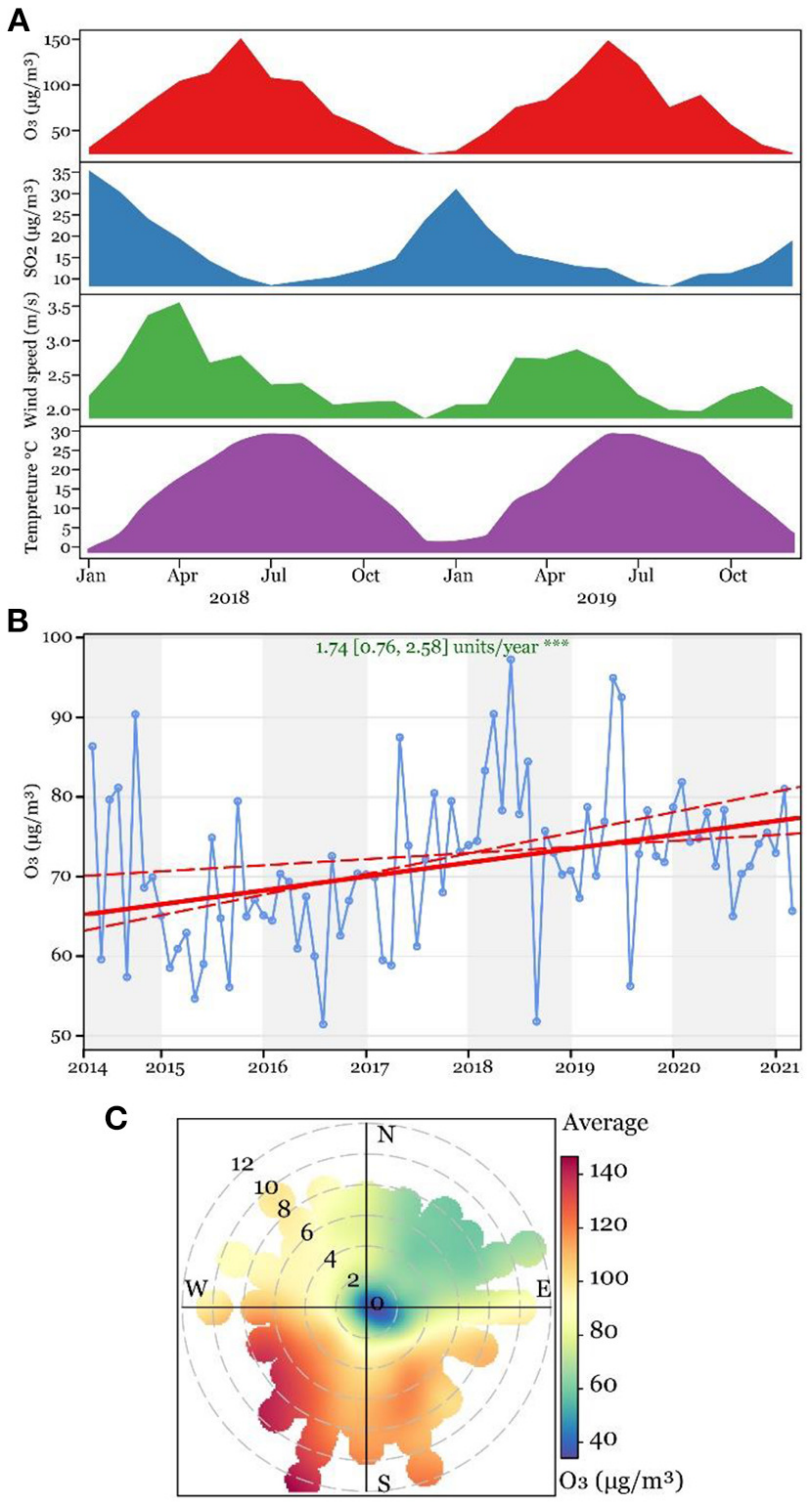
Variation of ground-level ozone in Jinan between 2014 and 2021. **(A)** Ridge plot of ground-level ozone, sulfur dioxide, wind speed, and temperature between 2018 and 2019. **(B)** Theil-sen trend analysis of ground-level ozone between 2014 and 2021. **(C)** Variation of ozone at dimensions of different wind speeds and directions.

As shown in Figure 1, the ambient ozone, sulfur dioxide (SO_2_), wind speed, and temperature changed periodically during the study period. For ozone and temperature, they were consistently higher in summer and lower in winter. In contrast, the ambient SO_2_ levels and wind speed changed in the opposite way. The average level of ozone was 74.00 ± 5.72 μg/m^3^, ranging from 57.59 to 91.05 μg/m^3^. The Theil-sen trend analysis showed that ambient ozone elevated from 2014 to 2021 by 1.74 μg/m^3^ each year. In combination with wind direction and speed, ambient ozone was visualized using a polar plot. As shown in Figure 1C, a higher ambient ozone level was identified when the wind from the south and southwest> 6 m/s, and a northern wind regardless of its strength corresponded to a low ambient ozone level. These data indicate that, unlike other air pollutants, ambient ozone in Jinan is positively correlated to the local temperature and shows an increasing trend over recent years.

### Baseline characteristics of study subjects

In total, 407 couples were involved in this study, they were 30.05 ± 3.60 years old on average, and 13.41 ± 1.02 years old at menarche, ranging from 19 to 42 and 10 to 18. The average BMI was 21.51 ± 2.99 kg/m^2^ (ranging from 15.24 to 35.76), the gestational age was 39.60 ± 0.99 weeks (ranging from 37 to 41.57), and the birth weight was 3408.00 ± 378.91 g (ranging from 2,105 to 5,150). The menstrual cycle of 18.18% of study subjects was irregular, and over 90% of women were educated at middle or higher levels. The occurrences of historical gestation, parity, and abortion were 41.77, 34.64, and 2.21%. Of the 407 newborns, 21 babies weighing >4,000 g were diagnosed with macrosomia. A total of 210 babies were boys, and 197 babies were girls, occupying 51.60 and 48.40% of total infants, respectively. For paternal factors, 19.41 and 17.20% of participants confirmed the consumption of cigarettes and alcohol.

According to birth weight, the cohort was divided into two groups: the macrosomia group and the non-macrosomia group. The differences in all demographical variables and ozone exposure levels were compared and shown in Table 1, among which the differences in maternal age, body height, age at menarche, menstrual status, maternal education background, historical gestation and parity, paternal smoking and alcohol consumption, gestational age, and fetal gender were of no statistical significance. Compared with the non-macrosomia group, the maternal pre-pregnancy weight, BMI, and fetal weight were statistically higher in the macrosomia group (*p* < 0.05), but the ambient ozone exposure level was lower and statistically significant at 0.10 level.

**Table 1:** Comparison of baseline characteristics between groups (*N* = 407).

**Variables**	**Non-Macrosomia**	**Macrosomia**	***P*-value**
	**(*N* = 386)**	**(*N* = 21)**	
Age (yrs)	30.01 ± 3.57	30.71 ± 4.12	0.452
Height (cm)	162.97 ± 4.43	163.14 ± 5.13	0.882
Weight (kg)	56.76 ± 7.95	64.29 ± 12.40	0.012
BMI (kg/m^2^)	21.36 ± 2.82	24.18 ± 4.58	0.011
Age at menarche (yrs)	13.42 ± 1.03	13.14 ± 0.73	0.107
Menstrual status			>0.999
Irregular	70 (18.13%)	4 (19.05%)	
Regular	316 (81.87%)	17 (80.95%)	
Maternal education background			0.218
Primary level	9 (2.33%)	1 (4.76%)	
Middle level	89 (23.06%)	7 (33.33%)	
Senior level	288 (74.61%)	13 (61.90%)	
Gestation			0.432
≥1	159 (41.19%)	11 (52.38%)	
0	227 (58.81%)	10 (47.62%)	
Parity			0.916
≥1	133 (34.46%)	8 (38.10%)	
0	253 (65.54%)	13 (61.90%)	
Paternal smoking			0.575
No	312 (80.83%)	16 (76.19%)	
Yes	74 (19.17%)	5 (23.81%)	
Paternal alcohol consumption			>0.999
No	319 (82.64%)	18 (85.71%)	
Yes	67 (17.36%)	3 (14.29%)	
Gestational age (weeks)	39.59 ± 0.99	39.86 ± 0.96	0.210
Fetal gender			>0.999
Male	199 (51.55%)	11 (52.38%)	
Female	187 (48.45%)	10 (47.62%)	
Fetal weight (g)	3363.85 ± 331.13	4219.52 ± 273.30	< 0.001
O_3_ (μg/m^3^)	74.13 ± 5.69	71.64 ± 5.98	0.077

### Association of birth weight and increased ozone exposure levels

Three statistical models were built to investigate the association of prenatal ozone exposure, birth weight, and macrosomia. Utilizing the binary categorized birth weight (macrosomia vs. non-macrosomia) as a dependent variable, the univariate logistic regression analysis was first adopted by enrolling the demographical variables and ambient ozone exposure data, one factor each time. As shown in Table 2, one unit increase in maternal pre-pregnancy BMI was found to increase the risk of macrosomia by 27.5% [odds ratio (OR) and 90%CI: 1.275 (1.15, 1.417)], and the increase of ambient ozone exposure level corresponded to 6.60% decreased risk of macrosomia [O_Rozone_ 90%CI = 0.934 (0.882, 0.992)].

**Table 2:** Univariate and multivariate logistic regression analysis showing predictors of macrosomia.

**Variables**	***N***	**Univariate analysis**		**Multivariate analysis**	
		***p*-values**	**Odds ratio (90% CI)**	***p*-values**	**Odds ratio (90% CI)**
Age (yrs)	407	0.382	1.055 [0.953;1.165]		
BMI (kg/m^2^)	407	< 0.001	1.275 [1.151;1.417]	< 0.001	1.251 [1.117;1.406]
Age at menarche (yrs)	407	0.216	0.729 [0.471;1.084]		
**Menstrual status**
Irregular	74		Reference		
Regular	333	0.916	0.941 [0.393;2.665]		
**Maternal education background**
Middle level	96		Reference		
Primary level	10	0.409	0.529 [0.179;2.748]		
Senior level	301	0.876	0.918 [0.32;2.162]		
**Gestation**
≥1	170		Reference		
0	237	0.315	0.637 [0.301;1.337]		
**Parity**
≥1	141		Reference		
0	266	0.733	0.854 [0.405;1.880]		
**Paternal smoking**
No	328		Reference		
Yes	79	0.602	1.318 [0.514;3.000]		
**Paternal alcohol consumption**
No	337	0.717	1.260 [0.486;4.177]		
Yes	70		Reference		
Gestational age (weeks)	407	0.212	1.349 [0.919;2.033]	0.033	1.906 [1.176;3.205]
**Fetal gender**
Male	210		Reference		
Female	197	0.941	0.967 [0.457;2.03]		
O_3_ (μg/m^3^)	407	0.056	0.934 [0.882;0.992]	0.029	0.912 [0.850;0.978]

**Figure 2:**
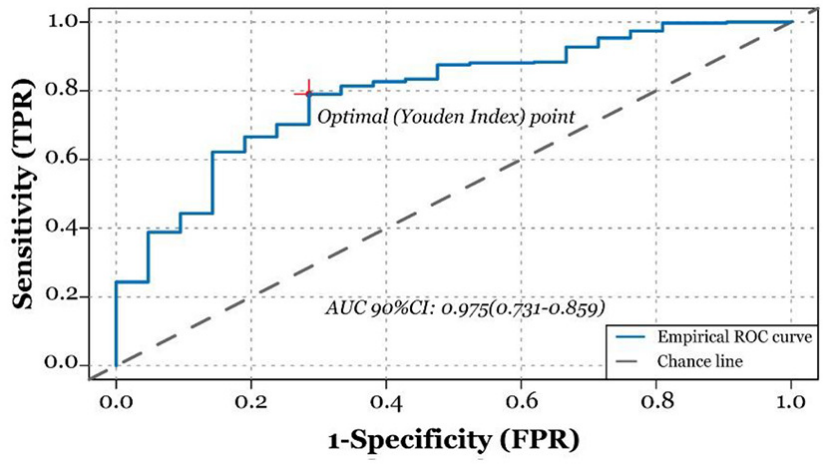
Receiver operating characteristic curve (ROC) analysis to test the robustness of the results of multivariate logistic regression model analysis. Area under the curve is 0.795 (90% CI: 0.716, 0.875).

To adjust the bias of demographical variables, we adopted the multivariate logistic regression analysis by simultaneously employing all demographical variables and ambient ozone exposure data. As shown in the right side of Table 1, BMI, gestational age, and ambient ozone exposure level were found to affect the occurrence of macrosomia at 0.05 level, and each unit increase of BMI and gestational age corresponded to 25.1 and 90.6% increased risk of macrosomia [OR_BMI_ 90%CI = 1.251 (1.117, 1.406); OR_Gestationalage_ 90%CI = 1.906 (1.176, 3.205)], while each unit increase of ambient ozone exposure decreased the risk of macrosomia by 8.80% [OR_ozone_ 90%CI = 0.912 (1.117, 1.406)]. The robustness of the multivariate logistic regression model was tested through ROC analysis in Figure 2, where we found the AUC and its 90%CI was 0.795 (0.716, 0.875). Also, a nomogram regarding fast-judging macrosomia was generated based on gestational week, ambient ozone exposure level, and BMI, shown in Supplementary Figure 1.

**Figure 3:**
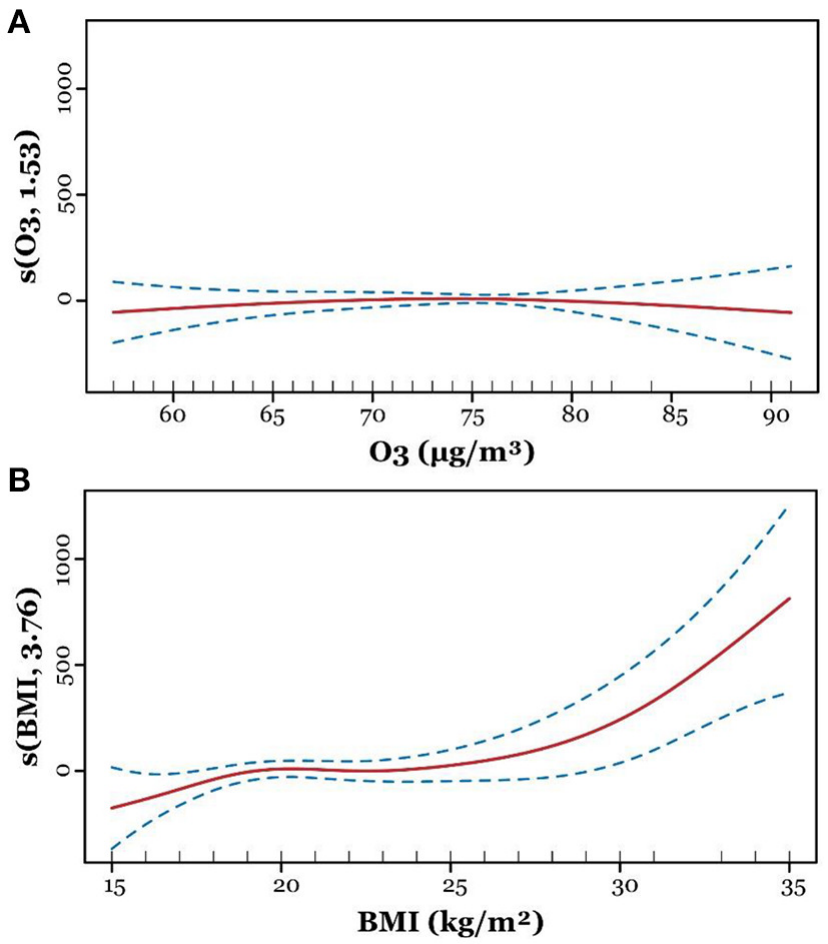
Adjusted generalized additive model for ozone and body mass index (BMI). After adjusting confounders, the line plots show smoothing functions with 95% confidence intervals for the association between birth weight and ozone **(A)** and BMI **(B)**.

The generalized additive model was built to study the linearity of ozone exposure level and birth weight, where continuous birth weight was involved as the dependent variable. By splining the ambient ozone exposure data and BMI, we found maternal age, gestational age, fetal gender, and the splined BMI could affect the increase in birth weight (Table 3). As shown in Figure 3, the birth weight increased with the elevation of BMI. Notably, a slight decrease in birth weight was observed with the increase of ambient ozone exposure, though the alteration was of no statistical significance, probably due to the limited sample size.

**Table 3:** Generalized additive model based on O_3_ and demographic variables associated with birth weight (*N* = 407)^a^.

**Variables**	**Estimate**	**Standard**	**t/F**	***p*-values**
		**error**	**value**	
Age (yrs)	11.228	6.067	1.851	0.065
Age at menarche (yrs)	−24.567	18.443	−1.332	0.184
Maternal education background	−4.689	22.678	−0.207	0.836
Menstrual status	−50.411	47.252	−1.067	0.287
Gestation	14.523	72.715	0.2	0.842
Parity	−42.01	80.012	−0.525	0.600
Gestational age (weeks)	101.398	19.55	5.187	< 0.001
Paternal smoking	21.169	49.692	0.426	0.670
Paternal alcohol consumption	9.772	51.018	0.192	0.848
Fetal gender	−92.465	36.628	−2.524	0.012
s(O_3_)			0.393	0.628
s(BMI)			4.039	0.002

^a^ O_3_ and BMI are included as smooth terms, the edf values of them are 1.533 and 3.765, and the ref.df values of them are 1.925 and 4.645.

## Discussion

Ambient ozone has been demonstrated as an emerging air pollutant that is associated with increased prevalence of various physical disorders, but few studies have been done regarding its effect on adverse pregnancy outcomes. Given the crucial role of birth weight that has been documented as an early life indicator for many childhood and adulthood diseases, we conducted this study primarily aiming to figure out the role of prenatal ambient ozone exposure on the decrease of birth weight and the occurrence of macrosomia as extreme conditions. We find that ambient ozone concentration is increasing over recent years, and prenatal exposure to increased ambient ozone is associated with decreased risk of macrosomia, but results of the multivariate generalized addictive model suggested a negative response of splined ambient ozone exposure on birth weight, but increased gestational age and splined pre-pregnancy BMI are found to cause a linear increase of birth weight.

The meteorology variation and climate change are factors affecting tropospheric ozone, and three dominant pathways have been defined by Lu's team, of which the natural emission of ozone precursors is affected by climate-sensitive natural sources such as lightening and biosphere, and the change of solar radiation, temperature, and humidity affect the chemical process of ozone generation and degradation (28). In line with previous studies, here we report the elevation of tropospheric ozone concentration in Jinan by 1.74 μg/m^3^ per year over the past 8 years, which should be an alarm for the increased physical disorders related to peroxidation in this area.

Pregnant women and fetuses are more vulnerable to environmental hazards such as ozone and particulate matter, but knowledge regarding the maternal and fetal effects of prenatal ozone exposure is limited. Previous studies indicate that ozone exposure can be linked to mortality and morbidity across the lifespan, including stillbirth, intrauterine growth restriction, preterm birth, and low birth weight (26, 29). Studies conducted among 1,125 pregnant women show that third trimester 8 h maximum ozone exposure is associated with an increased risk of adiposity at 5 months postpartum (30). Also, a systematic review shows a positive association of 1st and 2nd trimester ozone exposure with preterm birth (31), and each 10 μg/m^3^ increase in O_3_ exposure level causes an increased risk of low birth weight by 1.025 (95% CI: 1.020, 1.030) folds for the first trimester and 1.033 (95% CI: 1.028, 1.039) folds for second trimester (32). Consistent with previous studies, after adjusting potential confounders, we found a decreased risk of macrosomia with the elevation of ozone exposure concentrations by 0.912 (90%CI: 0.850, 0.978) folds. However, the splined ozone exposure concentration is not linearly correlated to birth weight, while a weak negative correlation between the two variables is found in Figure 3A, therefore their association should be further studied by expanding the sample size.

Exposure to different ambient pollutants has been demonstrated to cause adverse pregnancy outcomes. For example, based on the project Environmental and LifEstyle FActors iN metabolic health throughout life-course Trajectories (ELEFANT), situated in Tianjin, China, Juan Chen found that high levels of PM 2.5 exposure during pregnancy were associated with increased risk of preterm birth and low birth weight (33). In Australia, ShannonMelody reported that maternal exposure to even low levels of NO_2_ and PM 2.5 was associated with fetal growth restriction such as small for gestational age and term low birth weight (34). In our previous study, we also discussed the association between simultaneous various ambient pollutants exposure and preterm birth, and we find that, after adjusting the cofounding effects of demographical variables, NO_2_, CO, and SO_2_, maternal exposure to O_3_ increased risk of preterm birth [odds ratio 95% CI: 1.62 (1.19, 2.37), *P* < 0.05] (26).

## Conclusions

Based on the ambient ozone monitoring data between 2014 and 2021, we show the variation of ozone with wind speed and temperature between seasons and identified its increasing trend of 1.74 μg/m^3^ per year. After adjusting confounders primarily demographical variables, we find each unit increase in prenatal ozone exposure level causes 0.912 (90%CI: 0.850, 0.978) decreased risk of macrosomia, but the splined ambient ozone exposure level is not statistically associated with birth weight which is probably due to limited study population.

## Data availability statement

The raw data supporting the conclusions of this article will be made available by the authors, without undue reservation.

## Ethics statement

The studies involving human participants were reviewed and approved by Ethics Committee of Maternal and Child Health Care Hospital of Shandong Province. The Ethics Committee waived the requirement of written informed consent for participation.

## Author contributions

Project administration, methodology, data curation, and writing—original draft by CZ. Investigation and validation by JT. Validation by LM. Resources by CD. Conceptualization, resources, supervision, writing-review and editing, and funding acquisition by LZ. All authors contributed to the study conception and design, commented on previous versions of the manuscript, read, and approved the final manuscript.

## Funding

This work was financially supported by the Initial Scientific Research Fund of Young Talent in the Maternal and Child Health Care Hospital of Shandong Province (ISRFYT-2019-001). The funders had no role in study design, data collection, interpretation, or the decision to submit the work for publication.

## Conflict of interest

The authors declare that the research was conducted in the absence of any commercial or financial relationships that could be construed as a potential conflict of interest.

## Publisher's note

All claims expressed in this article are solely those of the authors and do not necessarily represent those of their affiliated organizations, or those of the publisher, the editors and the reviewers. Any product that may be evaluated in this article, or claim that may be made by its manufacturer, is not guaranteed or endorsed by the publisher.

## Supplementary material

Supplementary Figure 1 Nomogram generated based on the multivariate logistic regression analysis.
