# Supplementary figures and images for: Association between prenatal exposure to ambient ozone, birth weight, and macrosomia in healthy women
Source: Front Public Health. 2022 Nov 7;10:1000269. doi: 10.3389/fpubh.2022.1000269 (PMC9676959; doi:10.3389/fpubh.2022.1000269)

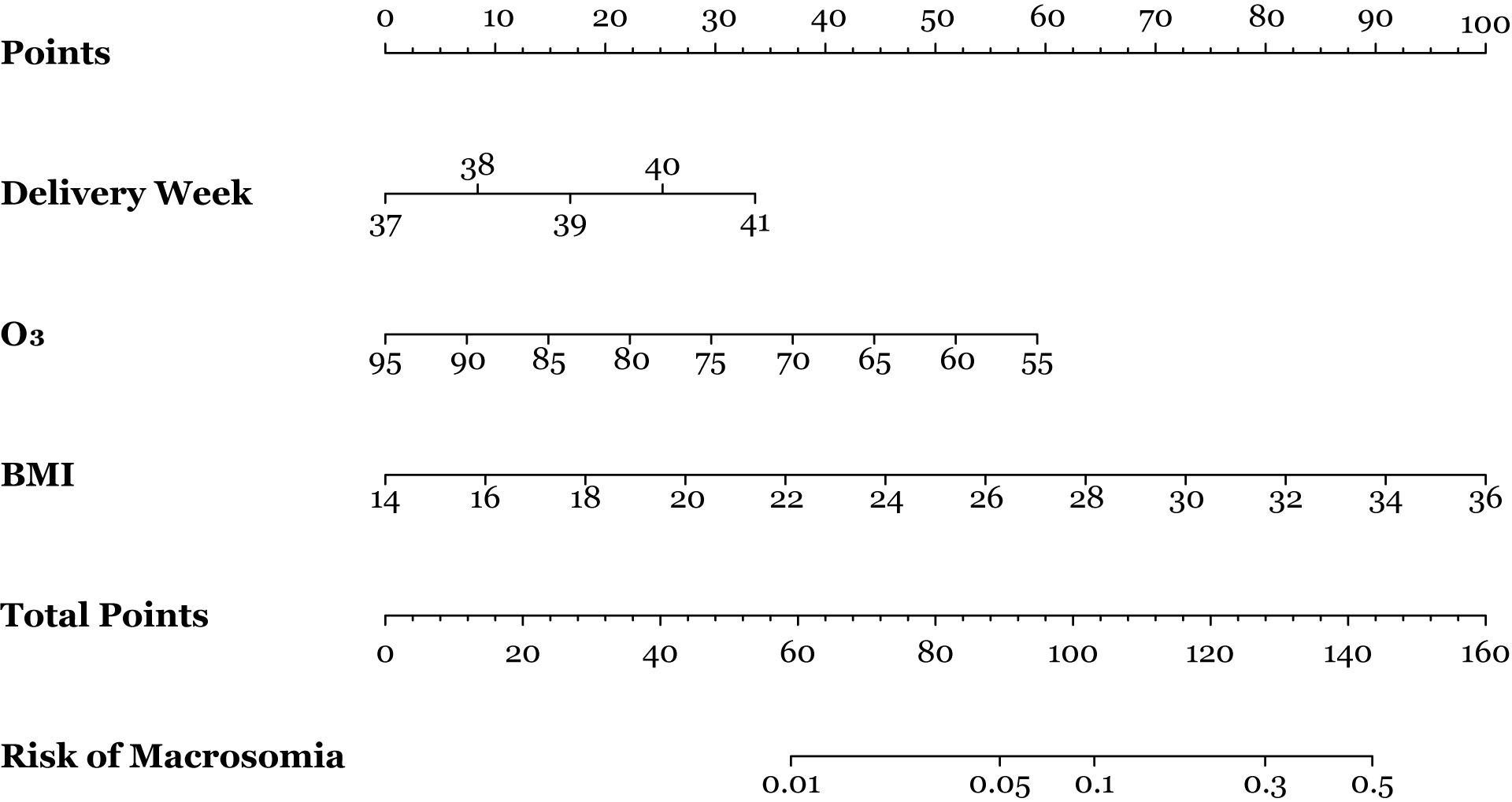

Supplement: Supplementary Figure 1 — Nomogram generated based on the multivariate logistic regression analysis. [file Image_1.JPEG]
